# A Randomized Trial Testing a Novel Mind and Body Intervention for Depression: Cognitive Behavioral Therapy (CBT) and Whole-Body Hyperthermia (WBH)

**DOI:** 10.1177/27536130251387714

**Published:** 2025-10-22

**Authors:** Ashley E. Mason, Wendy Hartogensis, Anoushka Chowdhary, Chelsea J. Siwik, Leena S. Pandya, Erika Jung, Osnat Lupesko-Persky, Erin Hartley, Lindsey Hopkins, Stefanie Roberts, Jenna Borovinsky, J. Craig Nelson, Christopher A. Lowry, Rhonda P. Patrick, Patricia J. Moran, Charles L. Raison, Frederick M. Hecht

**Affiliations:** 1 Osher Center for Integrative Health, 8785 University of California, San Francisco (UCSF), San Francisco, CA, USA; 2 Weill Institute for Neurosciences, 8785 University of California San Francisco (UCSF), San Francisco, CA, USA; 3 Department of Psychology, University of Arizona, Tucson, AZ, USA; 4 Department of Wellness and Preventive Medicine, 2569 Cleveland Clinic, Cleveland, OH, USA; 5 Department of Integrative Physiology, 1877 University of Colorado Boulder, Boulder, CO, USA; 6 FoundMyFitness, LLC, San Diego, CA, USA; 7 Department of Psychiatry, School of Medicine and Public Health, 5228 University of Wisconsin-Madison, Madison, WI, USA; 8 Vail Health Behavioral Health Innovation Center, Edwards, CO, USA; 9 Division of General Internal Medicine, 8785 University of California, San Francisco (UCSF), San Francisco, CA, USA

**Keywords:** major depressive disorder, whole-body hyperthermia, cognitive behavioral therapy

## Abstract

**Objective:**

To assess the acceptability of a randomized single-blind trial of cognitive behavioral therapy (CBT) and whole-body hyperthermia (WBH) treatment for major depressive disorder (MDD).

**Methods:**

All participants (N = 30) with MDD received CBT for depression and were randomized to also receive either: (1) WBH that raised core body temperature using an infrared sauna device, or (2) sham WBH of a similar duration that did not significantly raise core body temperature.

**Results:**

Study acceptability was the primary outcome: of participants who completed the final assessment (n = 29; 96.7%), 22 (75.9%) reported that they would recommend participation to a friend or family member with MDD. Twenty-five (86.2%) participants reported that they would be *likely* or *extremely likely* to enroll in this study, given the experience they had in the study. All participants randomized to WBH correctly believed they received WBH, and 6 (43%) of participants randomized to sham WBH correctly believed they received sham WBH. Both arms achieved clinically meaningful and statistically significant reductions in depression symptoms. The average decreases in the Beck Depression Inventory-II (BDI-II) were −19.07 (SE = 2.69, *P* < 0.0001) in the WBH arm (80.0% no longer meeting DSM-5 criteria, 60.0% achieving 50% or greater reduction in BDI-II) and −21.10 (SE = 2.41, *P*<0.0001) in the sham WBH arm (92.9% no longer meeting DSM-5 criteria, 78.6% achieving 50% or greater reduction in BDI-II).

**Conclusions:**

Study procedures were acceptable. Participants in the WBH and sham WBH groups had substantial reductions in depressive symptoms that were greater than typically seen with CBT alone. The sham WBH arm was not fully credible and may have exerted antidepressant effects, thus raising concerns about its use in future trials. Further research to test whether adding WBH to CBT results in additional antidepressant effects is warranted.

## Introduction

Major depressive disorder (MDD) affects 5% of adults (4% among men, 6% among women) worldwide and is a leading cause of life-years lost to disability.^1^ Currently available pharmacological treatments have significant limitations, including limited efficacy, delayed onset of action, and side effects that promote treatment non-adherence and/or discontinuation and impair quality of life.^2-4^ Currently available psychotherapeutic treatments are effective for some individuals but often do not provide sufficient symptomatic relief.^5^ Other treatments, such as electroconvulsive therapy and accelerated transcranial magnetic stimulation, have higher remission rates but pose financial and logistical obstacles.^6,7^ Such limitations have spurred interest in augmenting current treatment modalities with novel approaches that target different mechanisms that may underpin depression.

Several trials, including randomized controlled trials, have reported that whole-body hyperthermia (WBH) can elicit rapid reductions in depression symptoms.^8-10^ The rationale for using WBH as a treatment for depression is based on a growing body of observational and experimental studies suggesting associations between body temperature and MDD. A study of 20,880 adults reported that individuals with greater depressive symptomology had higher body temperatures assessed both via self-report and a wearable device.^11^ Smaller observational studies have similarly shown that body temperature is higher among individuals with depression,^12,13^ and experimental studies have found that temperature elevations among individuals with depression decrease upon clinical improvement.^14,15^ WBH targets thermoregulatory processes by acutely raising body temperature, thereby inducing sweating and subsequent cooling.^8,9,16^ Indigenous body heating practices spanning hundreds and thousands of years, such as sauna, hammam, and sweat lodge, have been used to promote physical and mental well-being.^17^ The diverse range of heat sources, practice durations, associated ceremonies, social dynamics, and other facets of these traditional practices highlight their evolutions within societies around the world. One common factor across these traditions, applying heat to an extent that is likely to increase body temperature such that it confers physiological, psychological, or other benefits, has received minimal scientific attention in the realm of mental health. To date, small pilot studies in laboratory settings using infrared heating devices, hyperthermic baths, or hot yoga have shown promise in improving mood in people with depression.^8,10,18,19^ Researchers have yet to elucidate whether there are specific WBH targets, such as core body temperature, WBH duration or frequency, or some combination, that confer maximal antidepressant effects, or if there are minimal doses that exert clinically meaningful effects.^9^ Our prior WBH depression protocols have focused on core body temperature,^8,10,20^ and we have built upon this by again focusing on a core body temperature target in this trial.

Rather than investigating WBH for depression alone, we assessed the acceptability of augmenting a first-line, evidence-based, efficacious treatment for depression: cognitive behavioral therapy (CBT). CBT addresses negative automatic thinking and cognitive distortions that the cognitive model of depression hypothesizes are central to depressive symptoms.^21^ CBT’s advantages over other psychotherapies include: (1) CBT can be administered in a time-limited manner (i.e., an eight-session protocol) by a diverse range of mental health clinicians, such as marriage and family therapists (MFTs), clinical social workers (CSWs), and other masters and doctoral level behavioral health clinicians, (2) CBT is manualized, with copious manuals, workbooks, and therapist guides available^22,23^; (3) there are validated protocols for ensuring and assessing clinician fidelity to CBT^24,25^; and (4) CBT is a first-line treatment for depression supported by meta-analytic data.^26,27^ Despite these strengths, a substantial proportion of people with MDD do not achieve sufficient depression relief from CBT alone and could benefit from adjunct treatment.^28^

In prior work,^20^ we augmented a manualized CBT protocol for depression with WBH sessions administered in the laboratory using an infrared heating device for adults with MDD. We found that the treatment was feasible and acceptable, and exploratory analyses indicated that among participants who completed treatment, 91.7% no longer met criteria for MDD at post-treatment, and that on average, participants experienced a 15.8-point reduction in the Beck Depression Inventory (BDI-II).^20^ In the current study, we sought to compare this treatment (WBH and CBT) to a treatment that included an identical CBT program but that included sham WBH sessions that did not significantly raise core body temperature. As we were principally focused on conducting this pilot RCT in preparation for a larger trial, our primary outcome was study acceptability; our secondary outcomes were pre-post treatment changes in depressive and related symptoms.

## Methods

### Study Design

This was a two-arm randomized clinical trial, conducted at an academic medical center, wherein participants were assigned to receive eight weekly sessions of CBT for depression and four biweekly WBH or sham WBH sessions. We designed this trial as such so that in a future efficacy trial, we would be able to assess the extent to which we can improve upon CBT outcomes by adding a body-based intervention. As this was a pilot trial, we did not power this study for the detection of an efficacy outcome. We chose a sample size of N = 30, which allowed us to have moderate precision in estimating our primary outcome (acceptability). If 25 of 30 Phase 2 participants rated the study experience as greater than 7 (83%), the 95% CI would be 70% to 97% (normal approximation to the binomial), which we believed would be adequate for this pilot phase of work. We used CONSORT-SPI reporting guidelines.^29^

### Participants

We enrolled participants in the San Francisco Bay Area between February 2023 and June 2024 (final data collection occurred in September 2024). We recruited participants via social media, posted flyers, university email marketing, and invitation letters delivered via the University of California, San Francisco (UCSF) electronic medical record system. Eligible participants were adults with a primary diagnosis of Major Depressive Disorder (MDD) based on a clinician-administered Structured Clinical Interview for DSM-5 (SCID-5-RV) and a BDI-II score of 21 or greater (exclusive of the suicide item) that did not decrease by more than 30% between two screening administrations at least 1 week apart (we included the BDI-II suicide item at the in-person screening and baseline visit, and all subsequent administrations). Eligible participants were not using antidepressant medications or medications with known effects on thermoregulatory processes, and met other inclusion and exclusion criteria as previously outlined. Exclusion criteria were primarily based on safety. For example, we excluded people who were pregnant or at risk of becoming pregnant, as WBH may negatively impact pregnant persons and/or fetal outcomes.^30^ We excluded individuals currently or recently (within the past 8 weeks) using antidepressant medications due to prior research: (1) suggesting that antidepressants impact thermoregulation^31^ and (2) data showing limited WBH-induced reductions in depression symptoms in these individuals.^8^

### Procedures

The UCSF Institutional Review Board (IRB) approved all study procedures (IRB# 21-34246). The UCSF IRB designated the WBH protocol, which used a commercially available WBH device, as posing non-significant risk (NSR), consistent with US Food and Drug Administration good guidance practices regulations (21 CFR 12). This research study was performed in accordance with the principles stated in the Declaration of Helsinki. This trial was registered on Clinicaltrials.gov (NCT05041361) before recruitment commenced.

#### Online and Phone Screening

All prospective participants provided electronic consent for study screening activities before completing an initial online screening survey. The initial screening survey included questions to determine initial eligibility, including the use of excluded medications and medical conditions,^20^ as well as the BDI-II (without the suicide question #9). Prospective participants who were eligible on the initial screening survey completed a phone screening during which study staff asked additional and more detailed eligibility questions (e.g., confirmed prospective participants’ self-reported medication and supplement use and medical conditions) and explained and answered questions about study procedures. Study staff then scheduled in-person screening and baseline visits for eligible and willing prospective participants. The study principal investigators reviewed each prospective participant’s medication and supplement use. To ensure stable depression symptoms, all prospective participants repeated the BDI-II (without the suicide item #9) the day before their in-person screening and baseline visit; those whose initial screening was more than 21 days before their in-person visit also completed the BDI-II 7-10 days before their in-person visit. Prospective participants with BDI-II scores less than 21 at these final screenings or whose scores dropped 30% or more between the final two BDI-II administrations were not eligible.

#### In-Person Screening and Baseline Visit

At the in-person screening and baseline visit, prospective participants completed the final set of screening activities, including self-report measures, anthropometric assessments, and (if female) a urine pregnancy test. A study mental health clinician completed a structured clinical interview to ensure prospective participant eligibility. The study mental health clinician verbally administered the Antidepressant Treatment Response Questionnaire (ATRQ)^32^ to further assess for recent or current antidepressant medication use and also assessed historical suicidality (past 12 months) using the Substance Abuse and Mental Health Services Administration (SAMHSA) suicidality item.^33^ To be eligible, a prospective participant’s primary diagnosis must have been MDD. If the prospective participant was eligible after completing all screening procedures, study staff reviewed the full study consent form with the prospective participant and answered any questions. Study staff reviewed several study procedures, including: explaining the potential to be randomized to receive either four WBH sessions or four sham WBH sessions (SWBH); that the study team would not be able to alter or disclose information about randomization; that participants would need to refrain from the following during the 10-week study period: using non-study sauna or heat practices; using alcohol or nicotine for 24 hours before and 24 hours after each heat treatment session; and vigorous exercise the day of each heat treatment session (WBH or SWBH). If prospective participants were eligible and agreed to these study procedures, they provided written consent.

#### Self-Report Measures

We administered all self-report measures (eg, online screening surveys, in-person self-report measures) online using Qualtrics survey software.^34^ We administered all in-person self-report measures on a dedicated study tablet.

#### Randomization

We randomized participants at the start of their first intervention visit. A biostatistician prepared a randomization sequence with a 1:1 ratio using a random command within Microsoft Excel. A blinded programmer embedded this sequence within the study database and study staff clicked on the allocation button for each participant to reveal their assigned study arm at the start of their first study visit.

#### Study Interventions

Participants received eight weekly CBT sessions with a study therapist and were randomized to receive either four bi-weekly WBH sessions or SWBH sessions (see below for study intervention details). We aimed to schedule participants’ CBT sessions weekly and WBH sessions biweekly; due to scheduling complexities, there were occasions when participants needed to reschedule study visits. Due to scheduling challenges, some participants began with a CBT visit first, and other participants began with a WBH or sham WBH visit first.

##### Study Intervention: Whole-body Hyperthermia (WBH)

As done in our single-arm trial,^20^ we provided WBH sessions using the Clearlight Curve Sauna Dome (Berkeley, CA), which is commercially available. In our initial research using this device,^35^ we found that it performed similarly (eg, required a similar amount of time to reach a core temperature of 38.5°C) as did the Heckel HT3000 hyperthermia machine in earlier studies.^8,8^ We used ancillary equipment (Mindray iPM-9800) to assess core (rectal) body temperature throughout WBH sessions (one temperature value per minute). Similar to how we have used other infrared devices in studies of WBH for depression,^10^ the Clearlight Curve Sauna Dome uses far-infrared heating elements. These heating elements are made of carbon fiber sheets and ceramic. In WBH sessions, the Sauna Dome ambient temperatures reached approximately 57.2°C, and we heated participants until they achieved a core body temperature of 38.5°C or until the maximum permitted WBH heating time had passed (140 minutes). Two study staff members remained with the participant during each WBH session: One study staff member sat on a chair near the participant’s head, applied cool cloths and ice to the participant’s head and neck, and provided the participant with electrolyte beverages and/or water to drink as often as the participant requested it. The second study staff member monitored the participant’s core body temperature on the Mindray iPM-9800, which was connected to an indwelling rectal probe that the participant privately inserted into their own rectum before the start of each WBH session. After a participant reached a target core body temperature of 38.5°C for two consecutive minutes, study staff removed the Sauna Dome and covered the participant in warm towels to allow gradual cooling over the next 30 minutes.

##### Study Intervention: Sham Whole-body Hyperthermia (SWBH)

SWBH sessions were identical in nature to WBH sessions with the following exceptions: The Sauna Dome ambient temperatures reached about 46.0°C and we heated participants only until they reached a target temperature of 0.1°C higher than their starting core body temperature. We designed the sham WBH for this trial to be maximally similar to WBH and to differ only in the core body temperature achieved.

##### Study Intervention: Cognitive Behavioral Therapy (CBT)

Four Doctorate- or Master-level licensed mental health clinicians provided eight weekly 50-minute CBT sessions. All clinicians had completed at least 1 year of CBT training in graduate school or as part of their work on other trials of CBT for depression, had at least 1 year of clinical experience practicing CBT in the community, at Veterans Affairs (VA) hospitals, or as providers in other clinical trials testing CBT for depression. The lead clinician who served as the primary clinician in our prior trial provided training to all study clinicians. Clinicians provided the standardized CBT for depression protocol, based on the Cognitive Behavior Therapy: Basics and Beyond, 3rd Edition,^23^ that we used in our single-arm trial.^20^ The CBT protocol focused on challenging dysfunctional cognitive processes and modifying behaviors.^22^ Participants completed CBT sessions via remote video sessions using Zoom, which data suggest to be as effective as in-person CBT.^36^ Mental health clinicians were blinded to participants’ assignment to WBH or SWBH.

#### Final Visit

After completing all intervention visits, participants attended a final visit during which they completed self-report measures and a study clinician re-administered the depression module of the clinical interview (SCID-5-RV) to assess for a diagnosis of MDD. A study staff member also interviewed each participant to learn more about the participant’s experience in the study by asking them if they had any feedback about the study interventions or assessments.

### Measures

#### Participant Characteristics

We report summary statistics (means and standard deviations, or counts and percentages) on participant characteristics, including demographic factors.

#### Primary Outcomes

##### Study Acceptability

The net promoter score^37^ is a single-item measure that has been adapted to evaluate satisfaction with healthcare.^37^ Participants rated, on a scale from 0 (*would not recommend*) to 10 (*would definitely recommend*), “*How likely would you be to recommend participating in this study to a friend or family member with depression?*” In addition to the net promoter score, we asked participants to rate, on a scale from 1 (extremely unlikely) to 5 (extremely likely), “*How likely would you be to enroll in this study given your experience in this study?*” Participants responded to both items at their final visit.

#### Secondary Outcomes

##### Beck Depression Inventory-II (BDI-II)

The 21-item BDI-II^38^ is a measure of depressive symptoms that assesses both mood and somatic symptoms of depression. Respondents respond to each item on a 4-point scale that ranges from 0 to 3. The BDI-II total score is computed as a sum of all items, with higher scores indicating greater depressive symptoms. A clinically meaningful reduction in BDI-II scores has been described as a 50% or greater decrease from an initial assessment, or a more moderate 17.5% decrease from an initial assessment^39^ and/or as a reduction of 3-9 points.^40^ Participants completed the BDI-II as part of their initial online eligibility screening as well as the night before their baseline visit (participants completed the baseline BDI-II suicide item #9 during the in-person screening and baseline visit, as a clinician was available to assess positive responses). We administered the full 21-item BDI-II at the beginning of each CBT study visit and at the final visit.

##### Patient-Reported Outcomes Measures (PROMIS) Profile-29

The PROMIS-29^41^ comprises 29 items that assess depression symptoms (4 items) and anxiety symptoms (4 items), as well as physical function (4 items), fatigue (4 items), sleep disturbance (4 items), ability to participate in social roles and activities (4 items), pain interference (4 items), and pain intensity (1 item). All items use a 5-point response scale, except for the single item for pain intensity, which respondents rate on an 11-point scale (0-10); the pain intensity item is used in a raw score form (without T-score conversion). We scored the 4-item measures using the scoring tables in the PROMIS-29 scoring manual, which is appropriate when there are no incomplete responses. Higher scores on the depression, anxiety, fatigue, sleep disturbance, and pain interference measures indicate poorer function (eg, worse depression, worse anxiety). Higher scores on physical function and ability to participate in social roles and activities indicate better function (eg, better physical function). A clinically meaningful reduction in a PROMIS measure is 3-4 points.^42^ We administered the PROMIS-29 at the baseline and final visits.

##### Patient-Reported Outcomes Measures Profile (PROMIS) Depression 8a

The PROMIS Depression 8a^41^ includes the 4 depression items that appear within the PROMIS-29 and 4 additional items. The PROMIS Depression 8a items are specific to depressed mood and not to several of the less specific symptoms that can be present with depression, such as disturbances in appetite, sexual function, sleep, or fatigue. To score this metric, we used the Health Measures Scoring Service for PROMIS Depression 8a. This scoring system can accommodate missing items by using response pattern scoring.^41,43^ We administered the Depression 8a at the baseline visit, weekly, and at the final visit.

##### Hamilton Depression Scale (HAM-D_6_)

The HAM-D_6_ is a 6-item self-report measure of depression that assesses the core items of depressive states^44^ and is psychometrically comparable to the HAM-D_17_.^45^ We administered the HAM-D_6_ at baseline, weekly, and at the final visit.

##### Structured Clinical Interview for DSM-5 (SCID-5-RV)

A study clinician administered the SCID-5-RV,^46^ which included assessments for MDD and other mental health disorders (eg, substance abuse, schizophrenia) at the baseline visit as part of eligibility screening. A study clinician re-administered the depression module of the SCID-5-RV at the final visit. These interviews served as our metric of the frequency of meeting DSM-5 criteria for MDD. Study clinicians were blinded to participants’ assignment to WBH or SWBH.

#### Additional Outcomes

##### Sham Whole-Body Hyperthermia (SWBH) Believability

In addition to pre-registered primary and secondary outcomes, in this trial, we sought to test a sham WBH condition for potential viability in a future clinical trial. As done in prior WBH research,^10^ participants self-reported their best guess of which treatment they received (SWBH vs WBH) at their final study visit.

##### Thermal Overshoot

As described above, we continuously monitored participants’ core body temperatures using a rectal thermometer during SWBH/WBH sessions. Under dynamic thermal conditions, thermal sensation anticipates body temperature changes and can initially exaggerate the thermoregulatory cooling response, a phenomenon called “thermal overshoot.”^47-49^ Thermal overshoot follows from the extent to which sensory neurons detect the rate of skin temperature change and send high-frequency signals to the brain.^50^ The brain, in turn, perceives the stimulus as more intense than its actual temperature and may amplify physiological and psychological responses.^51^ We assessed the extent to which each condition (SWBH/WBH) elicited thermal overshoot, defined in practice as going below one’s starting core body temperature by 0.1°C or more after beginning active heating, as thermal overshoot in this context holds the potential to elicit psychological responses.^16,52^

##### Cognitive Behavioral Therapy (CBT) Clinician Fidelity

We audio-recorded each CBT session. A PhD-level clinician with over 20 years of expertise in CBT for depression used the Cognitive Therapy Scale-Revised (CTS-R) to code one randomly selected session for half of the study participants. CTS-R scores range from 0 to 72, with a minimum competence standard of 36.

##### Whole-Body Hyperthermia (WBH) and Sham Whole-Body Hyperthermia (SWBH) Protocol Adherence

At each WBH/SWBH study visit, participants responded to questions about adherence to study instructions on a dedicated study tablet. Specifically, we asked participants if they had done any heat-related activities (eg, saunas, hot tubs) outside of the study, done any vigorous exercise prior to coming in for their WBH/SWBH visit, or had used alcohol or marijuana in the prior 24 hours.

##### Adverse Events

Before and after each WBH/SWBH session, participants answered several face-valid questions about symptoms that may have occurred in response to heat (e.g., lightheadedness, headache, nausea) using yes/no response options. Participants were also able to report any additional symptoms. If participants endorsed any symptoms, study staff members asked participants a series of follow-up questions that probed when participants began experiencing each symptom (i.e., before the WBH visit, during the WBH session, during cooldown, or after a 30-minute cooldown) and the current level of the symptom (ie, none, mild, moderate, severe, or very severe). If a participant’s symptoms did not resolve by the end of the study visit, study staff members followed up with the participant over the next days as needed to ascertain the timing of symptom resolution.

### Statistical Analysis

#### Primary Outcome

##### Study Acceptability

Our pre-specified primary outcome for this pilot trial was the net promoter score for the entire sample. In follow-up conversations with several participants about their experiences in the study, we learned that they indicated that in many cases, they would be unlikely to recommend participation in the study because their friends and family members use antidepressant medications, which were exclusionary for this study. We therefore considered responses on the item, “*How likely would you be to enroll in this study given your experience in this study*,” to be an additional metric of study acceptability. We used Fisher’s exact test to assess overall acceptability and acceptability by study arm and report binomial exact 95% confidence intervals (CIs).

#### Secondary Outcomes

##### Within-Arm Changes in Depression and Anxiety Metrics

As this was a pilot trial, we did not plan between-arm comparisons in our trial design or trial registration. Instead, our secondary outcomes included examining within-arm pre-post treatment (10-12 weeks later) changes for preliminary evidence of improvements in relevant clinical outcomes including the (1) BDI-II; (2) PROMIS Depression 8a; (3) HAM-D_6_ (4) PROMIS measures (eg, depression, anxiety); from the baseline visit to the final visit; the frequencies of (5) achieving a >50% reduction in BDI-II scores; (6) achieving a reduction in BDI-II threshold above which patients self-reported feeling better (versus not; >17.5%^39^); and (7) meeting DSM-5 criteria for MDD at post-treatment. For continuous outcome measures, we computed separate models of change from baseline to final assessment for each measure, with indicators for timepoint (baseline or final visit) and study arm, as well as their interaction, and a random intercept to account for the correlation of repeated measures.

#### Additional Outcomes

##### Sham Whole-body Hyperthermia (SWBH) Believability

We report the number and percentage of participants who found the sham believable (i.e., who endorsed at the end of their participation that they believed they had received the WBH condition).

##### Thermal Overshoot

We computed the frequencies of thermal overshoot during SWBH and WBH sessions, defining thermal overshoot as a participant going below their starting core body temperature by 0.1°C or more after beginning active heating. We used Fisher’s exact test to compare the frequency of thermal overshoot during WBH and SWBH sessions and report 95% binomial exact CIs.

##### Retention

We report on retention and adherence (means and percentages) and reasons for dropouts.

##### CBT Clinician Fidelity

We computed an average across all sessions to compute an overall fidelity score. We report on clinician fidelity to the CBT intervention as a mean and standard deviation on the CTS-R and report on where this score fell on the published range.

##### WBH/SWBH Protocol Adherence

We report on adherence to instructions given to participants about using non-study heat-related activities (eg, saunas, hot tubs), engagement in vigorous exercise prior to coming in for their WBH/SWBH visit, use of alcohol or marijuana in the 24 hours prior to coming in for their WBH/SWBH visit. We also report on several aspects of WBH/SWBH session completion, including the number of WBH sauna sessions that achieved the target temperature (38.5°C), the duration of WBH and SWBH sauna sessions, and the maximum temperature change from the lowest core body temperature achieved to the highest core body temperature achieved.

##### Adverse Events (AEs)

We tabulated all mild, moderate, and severe adverse events, and quantified the frequency of each type of reported event. We report these separately by treatment arm assignment (WBH or SWBH).

## Results

### Participant Characteristics

**Figure 1.**
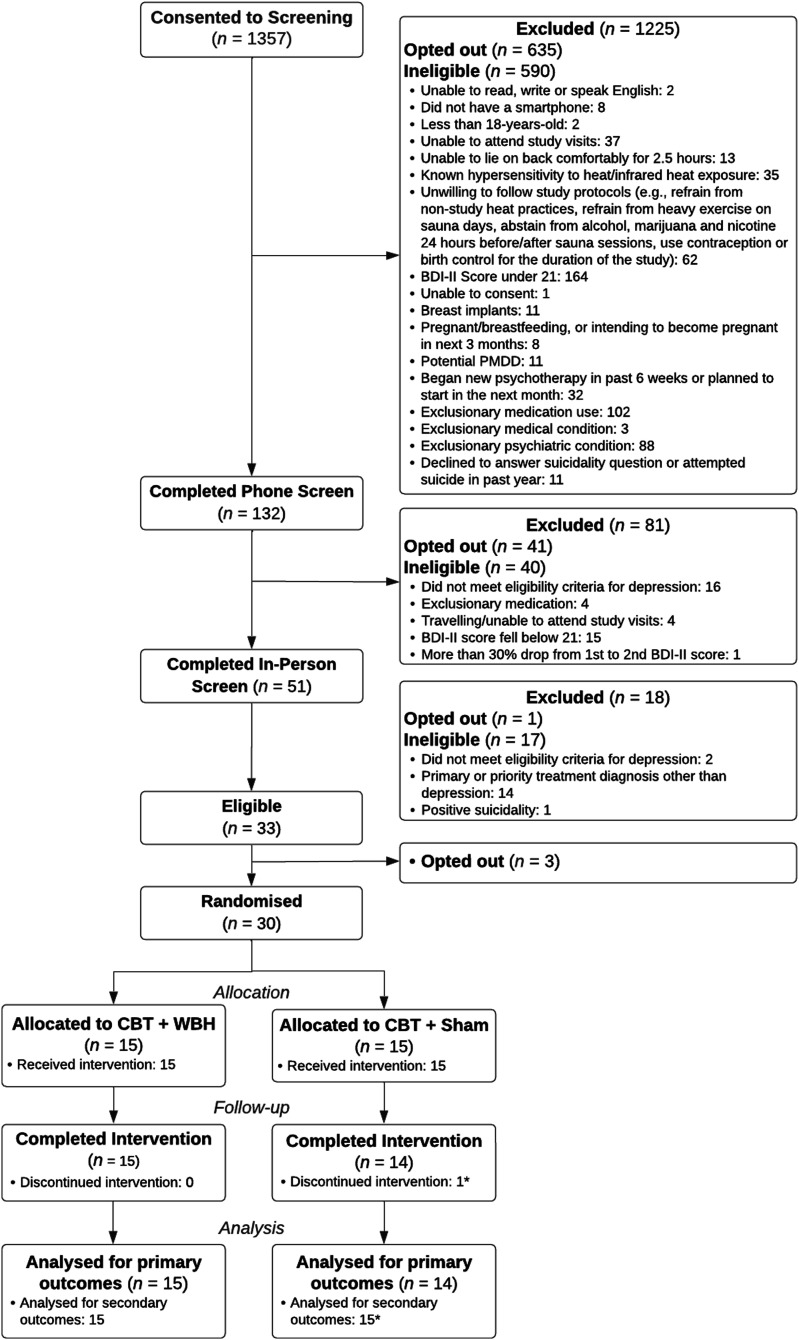
Participant Flow Through the Clinical Trial. *Note*. *One Participant Stopped Completing Sham WBH Visits and CBT Visits and did Not Complete the Final Assessment. The Participant Stated that They Were “Overwhelmed With Life” and Wished to Begin Using Antidepressant Medications

We enrolled 30 participants. Of 1357 individuals who consented to screening, 635 did not complete the screening process or did not respond to contact attempts (Figure 1). During the screening process, 41 individuals opted out of participation. Participants were 36.7% white, 23.2% male, and were on average 38.2 years old (Table 1). Of the 30 enrolled participants, 29 participants completed participation through the final assessment visit, which was completed an average of 10.9 weeks (SD = 2.5 weeks) after the baseline assessment (Figure 1).

**Table 1.** Participant Characteristics

Characteristic	Whole-body Hyperthermia (WBH), n = 15	Sham Whole-body Hyperthermia (SWBH), n = 15	Total Sample, N = 30
Age, years, M (SD)	40.47 (13.89)	35.87 (13.06)	38.17 (13.45)
Sex assigned at birth, N (%)			
Male	4 (26.7%)	3 (20.0%)	7 (23.3%)
Female	11 (73.3%)	12 (80.0%)	23 (76.7%)
Gender N (%)			
Man	3 (20.0%)	2 (13.3%)	5 (16.7%)
Woman	11 (73.3%)	12 (80.0%)	23 (76.7%)
Genderqueer	0 (0.0%)	1 (6.7%)	1 (3.3%)
Other^a^	1 (6.7%)	0 (0.0%)	1 (3.3%)
Race and ethnicity^b^ N (%)			
African American/Black	1 (6.7%)	0 (0.0%)	1 (3.5%)
Asian	1 (6.7%)	4 (28.6%)	5 (17.2%)
Latino/Hispanic	5 (33.3%)	4 (28.6%)	9 (31.0%)
Native American	0 (0.0%)	0 (0.0%)	0 (0.0%)
Pacific Islander	0 (0.0%)	0 (0.0%)	0 (0.0%)
White	7 (46.7%)	4 (28.6%)	11 (37.9%)
More than one race	1 (6.7%)	2 (14.3%)	3 (10.3%)
Education N (%)			
High school diploma or GED	0 (0.0%)	0 (0.0%)	0 (0.0%)
Some college	1 (6.7%)	1 (6.7%)	2 (6.7%)
Associate’s degree (eg, AA, AS)	1 (6.7%)	3 (20.0%)	4 (13.3%)
Bachelor’s degree (eg, BA, BBA, BS)	8 (53.3%)	5 (33.3%)	13 (43.3%)
Master’s degree (eg, MA, MS, MEng)	5 (33.3%)	3 (20.0%)	8 (26.7%)
Professional degree (for example: MD, DDS, JD)	0 (0.0%)	1 (6.7%)	1 (3.3%)
Doctoral degree (eg, PhD, EdD)	0 (0.0%)	2 (13.3%)	2 (6.7%)
BMI, kg/m^2^, M (SD)	27.12 (6.45)	25.93 (5.54)	26.53 (5.94)
Blood pressure (SBP/DBP), mm Hg, M (SD)	114.2/76.3 (14.2/8.6)	113.0/78.7 (17.2/8.6)	113.6/77.5 (15.5/8.5)
Beck depression inventory II (BDI-II), M (SD)	32.73 (9.07)	32.27 (7.76)	32.50 (8.30)

a Gender: one person categorized as “other” reported the following gender categories: Genderqueer, Man, Non-Binary.

b Race and Ethnicity: One person in the SWBH arm declined to respond to the race and ethnicity questions and is not included in denominators presented here. One person categorized as “other or more than one” race and ethnicity reported “Native Hawaiian or Other Pacific Islander” and “white” race; a second person in this category selected “other” race and in follow-up text reported “Middle Eastern” race; the third person in this category reported “Asian” and “white” race; all three people in this row reported no Hispanic or Latino ethnicity.

### Primary Outcome

#### Study Acceptability

The average net promoter score for the entire sample (N = 29) was 7.86 (SD = 2.08) on a scale from 0 (*would not recommend*) to 10 (*would definitely recommend*), with 75.86% (95% CI: 56.46%, 89.70%) of participants indicating a score of 7 or greater. Eleven of 15 WBH participants and 11 of 14 SWBH participants reported a score of 7 or greater (Fisher’s exact *P* = 1.0). As described above, this proportion may have been biased downward (not reflecting the true underlying positive endorsement of the study) as participants reported that they would be unlikely to recommend participation in the study to a friend or family member due to their friends’ and family members’ antidepressant medication use, which was exclusionary for this study. The average acceptability index for all participants who completed the final assessment (n = 29) was meaningfully higher, at 4.21 (SD = 0.94) on a scale from 1 (*extremely unlikely to enroll*) to 5 (*extremely likely to enroll*), with 86.21% (95% CI: 68.34%, 96.11%) of participants indicating that they would be “likely” or “extremely likely” to enroll in this study given their experience in this study. Twelve of 15 WBH participants and 13 of 14 SWBH participants rated enrollment likelihood as “*likely*” or “*extremely likely*” (Fisher’s exact *P* = 0.60).

### Secondary Outcomes

#### Within-Arm Changes in Self-Report Measures

Within-arm differences achieved statistical significance (Table 2), indicating that the WBH and SWBH participants experienced statistically significant improvements in depression and related symptoms (BDI-II, PROMIS Depression 8a, PROMIS-29 measures, HAM-D_6_), anxiety symptoms (PROMIS Anxiety 4a), and MDD diagnosis status (SCID-5-RV).

**Table 2.** Secondary Outcomes: Within-Arm Change From Pre-to Post-intervention Derived From Linear Mixed Models, With Separate Models for Each Outcome

	Measure	*M* (SE) baseline visit	*M* (SE) final visit	*M* diff (SE) (final – Baseline)	95% CI (LB, UB)
WBH arm (n = 15)	BDI-II	32.73 (2.30)	13.67 (2.56)	−19.07 (2.69)	(–24.34, −13.79)***
	HAM-D_6_^a^	10.42 (0.99)	4.00 (1.10)	−6.42 (1.30)	(–8.96, −3.87)***
	PROMIS-29				
	Depression 8A	64.83 (1.56)	54.04 (2.35)	−10.79 (2.66)	(–15.99, −5.58)***
	Anxiety 4A	63.09 (1.22)	55.85 (1.71)	−7.24 (1.63)	(–10.44, −4.04)***
	Fatigue 4A	67.01 (1.57)	53.43 (2.98)	−13.57 (2.58)	(–18.63, −8.51)***
	Sleep disturbance 4A	59.61 (1.92)	50.78 (1.69)	−8.83 (2.26)	(–13.25, −4.40)**
	Social role abilities 4A	40.99 (1.40)	54.03 (2.07)	13.04 (2.36)	(8.41, 17.67)***
	Physical function 4A	46.68 (1.62)	50.23 (2.06)	3.55 (2.23)	(–0.83, 7.92)
	Pain interference 4A	53.53 (1.80)	53.15 (2.26)	−0.39 (2.09)	(–4.48, 3.71)
	Pain intensity 1A	3.00 (0.25)	2.73 (0.46)	−0.27 (0.44)	(–1.14, 0.60)
SWBH arm (n = 15)^b^	BDI-II	32.27 (1.97)	11.16 (2.32)	−21.10 (2.41)	(–25.82, −16.39)***
	HAM-D_6_	11.07 (0.93)	3.91 (0.93)	−7.16 (1.12)	(–9.36, −4.96)***
	PROMIS-29				
	Depression 8A	65.62 (1.41)	54.08 (2.26)	−11.54 (2.84)	(–17.12, −5.97)***
	Anxiety 4A	64.45 (1.09)	56.93 (1.65)	−7.51 (2.06)	(–11.55, −3.48)**
	Fatigue 4A	64.39 (1.89)	51.14 (2.46)	−13.25 (2.49)	(–18.13, −8.38)***
	Sleep disturbance 4A	55.07 (1.18)	51.22 (1.77)	−3.85 (1.85)	(–7.47, −0.23)*
	Social role abilities 4A	42.55 (1.77)	52.69 (2.24)	10.14 (2.79)	(4.68, 15.60)**
	Physical function 4A	43.94 (1.89)	43.19 (1.53)	−0.75 (1.68)	(–4.05, 2.54)
	Pain interference 4A	51.61 (2.28)	48.64 (2.15)	−2.97 (1.86)	(–6.62, 0.68)
	Pain intensity 1A	2.53 (0.57)	1.98 (0.56)	−0.55 (0.51)	(–1.55, 0.44)

*Note*. BDI-II = Beck Depression Inventory-II; PROMIS = Patient-Reported Outcomes Measures; HAM-D_6_: Hamilton Rating Scale for Depression 6-item scale.

^a^note that due to a tablet administration error, two participants in the WBH arm did not complete the baseline HAM-D_6_.

^b^note that one participant in the sham arm was missing post-intervention data and thus only contributed baseline data to the models reported here. *P < .05; **P < .001; ***P < .0001.

#### Clinically Meaningful Changes in BDI-II

The frequency of achieving a clinically meaningful improvement in BDI-II scores (>50% reduction) was 60.0% (9/15; 95% CI: 32.3%, 83.7%) in the WBH arm and 78.6% (11/14; 95% CI: 49.2%, 95.3%) in the SWBH arm. The frequency of achieving a smaller improvement in BDI-II scores (>17.5% reduction) was 86.7% (13/15; 95%CI: 59.5%, 98.3%) in the WBH arm and 92.9% (13/14; 95%CI: 66.1%, 99.8%) in the SWBH arm. Of note, all participants (n = 29) achieved a reduction of 3 or more BDI-II points between their baseline assessment and the final BDI-II assessment they completed (mean reduction across all 29 completers = 20.03 points, SD = 9.89).

#### Frequency of Meeting DSM-5 Criteria for MDD (SCID-5-RV)

The frequency of no longer meeting a diagnosis for MDD at post-treatment was 80.0% (12/15; 95%CI: 51.9%, 95.7%) in the WBH arm and 92.9% (13/14; 95%CI: 66.1%, 99.8%) in the SWBH arm.

### Additional Outcomes

#### Sham Believability

Among participants assigned to the WBH arm, 100% correctly believed they were in the WBH arm. Among participants assigned to the SWBH arm, only 42.9% (6 of 14 who completed a final survey) correctly believed that they were assigned to SWBH intervention, and the remaining 57.1% (8 of 14) incorrectly believed that they were assigned to the WBH arm.

#### Thermal Overshoot

Of 57 SWBH sessions, 54 (94.7%, 95%CI: 85.4%, 98.9%) evidenced thermal overshoot; of 53 WBH sessions, 52 (98.1%, 95%CI: 89.9%, 100.0%) evidenced thermal overshoot (Fisher’s exact *P* = 0.62).

#### Retention

Twenty-nine (96.7%) participants completed the final assessment. The participant who dropped out and did not complete the final assessment completed 3 CBT sessions and 1 WBH session. Notably, this participant’s change in BDI-II scores from baseline to the last data collection (4.4 weeks post-baseline) was 6 points (an 18.2% reduction over 31 days, from a score of 33 to a score of 27). Thus, even the one participant who dropped out of the study had experienced reduced depression symptoms at the time of their discontinuation.

### Cognitive Behavioral Therapy (CBT) Clinician Fidelity

All 30 participants completed at least three CBT sessions; 28 participants (93.3%) completed all 8 assigned sessions. The average total CTS-R score was 63.6 (SD = 5.5), indicating ratings in the *Expert* range (60-72) in delivering CBT per protocol (the minimum competence standard is 36).

#### WBH/SWBH Protocol Adherence

All 30 participants completed at least one WBH/SWBH session; 26 participants (86.7%) completed all four sessions.

##### Pre- and Post-WBH/SWBH Protocol Adherence

Two participants in the WBH arm reported non-adherence to the treatment (one used marijuana in the 24 hours before their first WBH session; the other reported outside sauna use 4 days before their third WBH session). One participant in the SWBH arm reported outside sauna use 11 days before their third SWBH session. No participants reported vigorous exercise or alcohol use in the 24 hours before a WBH/SWBH session.

##### WBH Sessions

Across all 53 WBH sessions, a participant’s body temperature increased to an average maximum of 38.45°C (SD = 0.42°C), with a median high of 38.60°C. The mean core body temperature increase from the lowest core body temperature achieved to the highest core body achieved was 1.67°C (SD = 0.40°C; median = 1.70°C). Of 15 participants randomized to the WBH arm, 12 participants achieved a core body temperature of 38.5°C in at least one WBH session. The average WBH session duration was 98.5 minutes.

Of the 53 WBH sessions, 38 reached the target body temperature (38.5°C). Among these 38 sessions, body temperature remained at or above 38.5°C for an average of 13.9 minutes (SD = 12.4 minutes) after the sauna was turned off before decreasing, and the median high core body temperature was also 38.60°C. Among these 38 sessions, the mean core body temperature increase from the lowest core body temperature achieved to the highest core body achieved was 1.83°C (SD = 0.26°C, median = 1.85°C). On average, WBH sessions reaching 38.5°C included 99.8 minutes of active heating.

Among the 15 (of 53) sessions that did not reach 38.5°C, the mean high core body temperature was 37.94°C (SD = 0.44°C) with a median high of 38.20°C. Among these 15 sessions, the mean core body temperature increase from the lowest core body temperature achieved to the highest core body achieved was 1.27°C (SD = 0.40°C, median = 1.30°C). On average, WBH sessions not reaching 38.5°C included 95.8 minutes of active heating.

##### SWBH Sessions

On average, a participant’s body temperature increased 0.1°C above where they began. The mean increase from the lowest core body temperature achieved to the highest core body temperature achieved was 0.45°C (SD = 0.12°C, median = 0.40°C), reflecting that on average, body temperatures decreased before increasing. On average, SWBH sessions were 74.9 minutes.

#### Adverse Events (AEs)

No serious AEs occurred in this study. Across the 53 active WBH sessions and the 57 SWBH sessions, participants reported several mild adverse events that are expected in heat therapies (Table 3). The most common mild AE in both arms was headache, followed by lightheadedness and nausea. In the WBH arm, three participants reported 8 or more AEs across all of their WBH sessions, three others reported 5-7 AEs across all sessions, and all other participants reported 4 or fewer AEs across all of their WBH sessions, with one participant reporting no AEs across 4 completed WBH sessions. In the SWBH arm, six participants reported 1-4 AEs across all sessions, and the remaining nine participants did not report any AEs across all of their SWBH sessions.

**Table 3.** Adverse Events (AEs)

AE	# AEs	# Participants reporting AE	Related to study intervention/Expected?/# AEs	*N* (%) AEs resolved by end of WBH visit	*N* (%) AEs resolved by end of WBH day
Whole-body hyperthermia (WBH)					
Headache	28	13	Definitely/Yes (28)	3 (10.7%)	25 (89.3%)^a^
Lightheadedness	15	7	Definitely/Yes (15)	4 (26.7%)	11 (73.3%)^a^
Nausea	8	5	Definitely/Yes (7)	2 (25.0%)	6 (75.0%)^a^
			Probably/Yes (1)		
Migraine	2	1	Definitely/Yes (2)	0 (0%)	2 (100.0%)^b^
Fatigue	2	1	Possibly/Yes (2)	0 (0%)	2 (100.0%)^a^
Vomiting	1	1	Definitely/Yes (1)	0 (0%)	1 (100.0%)
Bruising	1	1	Definitely/Yes (1)	0 (0%)	1 (100.0%)^b^
Bad sleep	1	1	Possibly/No (1)	0 (0%)	1 (100.0%)^b^
Head pressure	1	1	Probably/Yes (1)	0 (0%)	1 (100.0%)
Chills	1	1	Probably/Yes (1)	0 (0%)	1 (100.0%)
Stomach pain	0	-	-	-	-
Dry mouth	1	1	Possibly/No (1)	0 (0%)	1 (100.0%)^b^
Flu	0	-	-	-	-
Knee surgery	0	-	-	-	-
Sham whole-body hyperthermia (SWBH)					
Headache	6	3	Definitely/Yes (1)	1 (16.7%)	5 (83.3%)
			Probably/Yes (1)		
			Unlikely/Yes (4)		
Lightheadedness	3	2	Definitely/Yes (3)	1 (33.3%)	2 (66.7%)
Nausea	2	2	Definitely/Yes (2)	2 (100.0%)	0 (0%)
Migraine	0	-	-	-	-
Fatigue	0	-	-	-	-
Vomiting	0	-	-	-	-
Bruising	0	-	-	-	-
Bad sleep	0	-	-	-	-
Head pressure	0	-	-	-	-
Chills	0	-	-	-	-
Stomach pain	1	1	Unlikely/Yes (1)	0 (0%)	1 (100.0%)^b^
Dry mouth	0	-	-	-	-
Flu	1	1	Unlikely/No (1)	0 (0%)	1 (100.0%)^c^
Knee surgery	1	1	Not related/No (1)	0 (0%)	1 (100.0%)^c^

^a^Two episodes resolved by the next day.

^b^One episode resolved by the next day.

^c^One episode resolved by 7 days after the session.

Rates of adverse events were comparable between the n = 6 participants who believed (correctly) they were assigned to the SWBH arm and the n = 23 who believed they were assigned to the WBH arm (n = 23 includes the n = 15 who guessed correctly and the n = 8 who incorrectly believed they were assigned to the WBH arm).

## Discussion

The primary aim of this trial was to assess the acceptability of participating in a study evaluating a CBT and sham-controlled WBH treatment for major depressive disorder. Secondary aims included assessment of changes in depressive and related symptoms and the believability of a sham WBH comparator arm. We successfully recruited and enrolled 30 adults with depression, surpassing our projected retention rates, with 29 of 30 having completed the final assessment. Moreover, all participants experienced a reduction in depression symptoms by the time they finished their participation (even if they ended participation before the final visit). Notably, 25 of 29 participants (86.2%) no longer met criteria for MDD at the final visit, and the mean BDI-II decrease was 20.03 points across both arms.

For several reasons, little is known about whether there are optimal WBH targets (e.g., core body temperature, WBH duration or frequency, or some combination of targets) that confer maximal antidepressant effects or minimum doses that have clinically meaningful effects.^9^ First, prior studies have used varied body temperature assessment methods, rendering it difficult to compare results across studies. For example, studies have assessed body temperature from the rectum, sublingual area, and the ear. Second, prior studies have administered varied body heating protocols. For example, protocols have varied by body heating modality (e.g., hot tubs, infrared body heating devices) and durations of heating (e.g., a specified number of minutes vs. any amount of time needed to achieve a particular body temperature level or size of body temperature increase). The heterogeneities among body temperature measurement and body heating protocols and the lack of studies that directly compare different temperature targets limit our knowledge about optimal targets in WBH.

The uncertainty about the effects of different body heating protocols limits our knowledge of whether our sham heating protocol was actually inert. The SWBH condition lasted an average of 74.9 minutes, and prior WBH studies have found that body heating for this duration of time can confer antidepressant effects.^9^ We observed very high rates of thermal overshoot in both conditions (SWBH = 94.7%; WBH = 98.1%), which may partially explain similar changes in clinical outcomes. In thermal overshoot, thermoreceptors increase their firing rates, sending additional high-frequency impulses to the brain that can amplify physiological and psychological responses.^51^ The lower heat stimulus used in the sham condition appears to have induced thermal overshoot and sweating (body cooling) sufficient to maintain a lower core body temperature. As the afferent neural signaling from the cutaneous thermoreceptors to the lateral parabrachial nucleus induce thermoregulatory cooling,^53^ and the afferent neural signaling from the cutaneous thermoreceptors, through the mediodorsal thalamus and to the medial orbitofrontal context likely alter mood and cognitive function, it is possible that stimulating these cooling responses might confer some of the antidepressant effects of WBH.^16,54^ This fits with some of the theoretical pathways through which WBH may improve depression.^16,52^ The explanation for the apparent paradox of how WBH improves depression despite further increasing body temperature is hypothesized to be that it stimulates improved thermoregulatory responses, including improved cooling responses. Additionally, it is possible that the duration of time one is exposed to the heat stimulus could represent one key WBH target when administering WBH for MDD. Of the participants in the SWBH condition, 57% believed they received (actual) WBH. That we observed thermal overshoot in the SWBH condition demonstrates that there was an afferent thermoregulatory response.

This pilot trial aimed to assess acceptability of our protocol and was not powered or designed to compare effects between arms. With this critical caveat, our preliminary within-arm comparisons suggest the possibility of an antidepressant effect of both arms that may be greater than typically expected with CBT alone. The average BDI-II decreases in the SBWH (21.10 points) and WBH (19.07 points) conditions, and the rates at which participants in the SWBH (13/4; 92.9%) and WBH (12/15; 80.0%) conditions no longer met criteria for MDD, were much larger than those observed in meta-analyses assessing effects of CBT for depression. For example, one meta-analysis^55^ of 70 eligible trials included primarily within-arm studies (and some between-arm studies that included inactive waitlist control arms), and 67 of these 70 trials included BDI and BDI-II data. Data indicated an average within-arm effect size of CBT was Hedge’s *g* of 1.58, which translates to 6.5 BDI points, and an MDD remission rate of 57%. Notably, the average number of CBT sessions of studies included in this meta-analysis was 14.6 sessions, which is nearly 83% more CBT treatment than we provided in this study (8 CBT sessions). Data on the dose-response association are complex due to heterogeneity in how “dose” is defined (eg, total number of sessions, total contact time with a therapist, number of sessions per week).^56^ Data suggest that although the largest reductions in depression symptoms often occur early in treatment, larger psychotherapy doses are associated with greater reductions in depressive symptoms.^57^ This suggests that if the antidepressant effects in our trial were only due to CBT, we would have observed smalle\r effects. Instead, we observed antidepressant effects of combining CBT with WBH treatment in our prior single-arm study that were more than 2x as large^20^ and in the current study were nearly 3x as large. Together, these findings support the possibility that the WBH and SWBH arms both provided antidepressant effects.

This study tested two treatments that are now protocolized and manualized, and have systems for assessing fidelity (e.g., CBT sessions can be coded using a validated fidelity scale,^24,25^ WBH can be administered so as to ensure its comparability to prior studies^8,10,20,35^) for future testing in a larger randomized design. We devised techniques to reduce cost and burden for both providers (e.g., research staff, mental health clinicians) and individuals participating in future studies: For example, we tested a “flipped” model wherein participants presented in person for an initial screening, but the mental health clinician administering the screening clinical interview was present on Zoom. We also administered all CBT sessions over Zoom video conference. Together, these two design parameters allowed us to cast a wide net when building our mental health clinician team. We retained 96.7% of participants (n = 29 of 30) for our final assessment, suggesting that a depression treatment study design combining CBT and WBH can achieve excellent retention.

### Limitations and Future Directions

In addition to our limited sample size, an important limitation is that we did not have a direct comparison arm that received CBT alone. It is possible that the observed (high) response rates are attributable to factors such as highly effective therapists in an academic setting or chance rather than any effects of our heat treatments. Results from this trial raise concerns about using a heat-based sham condition in future trials testing WBH interventions. Future research may benefit from using a credible sham that does not provide heat, which would avoid including a potentially active heat treatment as the intended control condition. Additionally, future research would benefit from assessing participant perceptions of WBH (e.g., intensity and pleasantness of the heat). Because the duration and intensity of heat needed to avoid treatment effects are unknown, it is difficult to ensure an inert sham heat arm. This study also revealed challenges with fully masking research participants to heat treatment assignments: The sham condition was not uniformly credible, as nearly half the participants in the sham arm correctly guessed they were receiving sham treatment. As an example of a potential alternative comparison arm, a prior trial told participants that “two promising treatments are being compared” and found that hyperthermic baths (active treatment) were superior to green-light therapy (control) in reducing depressive symptoms among individuals with moderate depression.^19^ This approach did not rely on masking (i.e., participants knew which treatment they were receiving) and therefore may have obviated issues with ensuring inertness. Future trials may benefit from using a credible comparator condition that does not include body heating and ensuring that all participants receiving both WBH and CBT begin with the same treatment visit (e.g., WBH first or CBT first). We plan to conduct a 2 × 2 factorial trial assessing multisite feasibility in preparation for larger multisite efficacy testing that will omit a sham WBH condition. Specifically, we will administer CBT (yes/no) and WBH (yes/no) so that we can begin to separate the effects of CBT from WBH and explore the extent to which their combined effects may be additive or synergistic.

## Data Availability

The data that support the findings will be available at OSF (https://osf.io/), together with a data dictionary, upon publication.
